# Intellectually disabled patients’ intensive care admission characteristics, weaning from mechanical ventilation, and sedative drug use: a single-center retrospective case-control study

**DOI:** 10.1186/s44158-022-00081-4

**Published:** 2022-12-21

**Authors:** Noa Bineth, Nevo Barel, Tali Bdolah-Abram, Philip Levin, Sharon Einav

**Affiliations:** 1 Department of Military Medicine and “Tzameret”, Faculty of Medicine, Hebrew University of Jerusalem, Jerusalem, Israel; 2 Faculty of Medicine, Hebrew University of Jerusalem, Jerusalem, Israel; 3 General Intensive Care Unit, Shaare Zedek Medical Center and Hebrew University Faculty of Medicine, Jerusalem, Israel

**Keywords:** Intellectually disability, Mental-retardation, Critical-care, Airway-management, Respiration, Artificial, Medicine, Clinical

## Abstract

**Background:**

Intellectually disabled (ID) patients present unique therapeutic challenges. We aimed to describe the characteristics of ID patients admitted to a general intensive care unit (ICU).

**Results:**

We conducted a retrospective cohort study comparing critically ill adult ID patients to matched patients without ID (1:2 ratio) in a single ICU (2010–2020). The main outcome measure was mortality. Secondary outcomes included complications during admission and characteristics of weaning from mechanical ventilation. The study and control groups were randomly selected based on similar age and sex. ID patients nonetheless had an average APACHE score of 18.5 ± 8.7 vs. 13.4 ± 8.5 in controls (*p* < 0.001). ID patients had more hematological (*p* = 0.04), endocrinological (*p* < 0.001) and neurological (*p* = 0.004) comorbidities and used more psychiatric medication before admission. No difference was found in mortality rates. Differences were found as there were more secondary complications, such as pulmonary and sepsis (*p* < 0.03), frequent requirement of vasopressors (*p* = 0.001), significantly higher intubation rates with more weaning attempts, tracheostomies and longer ICU and hospital admissions (*p* < 0.019).

**Conclusions:**

Critically ill adult ID may have more comorbidities and be sicker at the time of admission compared to their age- and sex-matched counterparts. They require more supportive treatment and their weaning from mechanical ventilation may be more challenging.

**Supplementary Information:**

The online version contains supplementary material available at 10.1186/s44158-022-00081-4.

## Introduction

Intellectually disabled (ID) patients present unique therapeutic challenges. They demand more medical resources than the general population [1] and have higher rates of hospital and intensive care unit (ICU) admission [2]. Few studies exist regarding the characteristics of these patients and the therapeutic challenges they present in the ICU.

Intellectual disability is defined by the presence of cognitive ability limitations (meaning an IQ lower than 70) and difficulties in societal adjustment [3]. The etiologies of ID include environmental as well as genetic factors (for example chromosomal aberrances, single gene mutations) [3]. The worldwide reported of prevalence of ID is 2–3% and the population of patients with ID is increasing [3]. Among ID patients, many have Down Syndrome (DS) [4].

ID patients suffer lifelong disability and early mortality [4, 5]. Compared to the general population, they undergo more hospitalizations [2] and when hospitalized have longer lengths of in-hospital stay [4, 6]. They are admitted more frequently after surgical procedures, have more complications [7, 8] and are also more likely to die during hospitalization [9, 10]. Moreover, three of every four patients with ID receive chronic psychoactive drug therapy and half are treated with more than one medication [11]. Weaning un-cooperative adults from mechanical ventilation presents unique difficulties. Despite this, there is a paucity of clinically relevant medical literature regarding the ICU treatment and outcomes of patients with ID.

We aimed to describe the characteristics of ID patients admitted to a general ICU and to compare the rates of unadjusted, all-cause, ICU and in-hospital mortality of these patients to an age- and sex-matched cohort without ID. We also studied the duration of hospital and ICU stay, secondary complications, the process of weaning from mechanical ventilation, the medications used to assist the weaning process and readmission rates. We hypothesized that mortality rates, complication rates, and admission and ventilation durations are greater in ID patients than in controls and that these patients receive higher doses of sedative medication in the days before extubation.

## Methods

### Study design

This retrospective, case-control study included patients with and without intellectual disability (ID) who were admitted to a single general intensive care unit (ICU) in Israel. The study was approved by the institutional review board (0085-19-SZMC) with waiver of informed consent and is reported according to STrengthening the Reporting of OBservational studies in Epidemiology (STROBE) recommendations [12].

### Setting

The Shaare Zedek Medical Center (SZMC) is a tertiary hospital with > 1000 adult acute care beds, > 50 intermediate care beds and 14 general ICU beds. In the general ICU, triage, extubation, and discharge decisions are made by an ICU team which always includes at least one boarded intensivist. Approximately 650 patients are treated in the general ICU annually.

### Participants

All adult patients (≥ 18 years) admitted to the SZMC general ICU (2010-2020) were eligible for study inclusion. The study (ID) group included patients with at least one of the following three International Classification of Disease (ICD) admission diagnoses in their electronic medical record (EMR): mental retardation (ICD-9 code 317-319), Down syndrome (ICD-9 code 758.0) or disorder of pituitary gland (ICD-9 code 253). Following preliminary inclusion, patient files were manually screened to ensure fulfillment of inclusion/exclusion criteria and to confirm the presence of an ID.

The control group included non-ID patients admitted to the ICU during the same study period with a similar age and sex. For each ID patient two non-ID patients were randomly selected using the free online Python random module (see Supplement 1). If no matching control patient was found, patients of the same sex but with a single year of age difference were matched.

For both study and control patients, if more than one ICU admission occurred during the study period, we analyzed only the data from the first admission but documented the occurrence of a second hospitalization with ICU admission.

### Variables

The primary study outcome measures were the rates of unadjusted, all-cause, ICU, and in-hospital mortality in the study and control groups. The secondary outcome measures included comparative population characteristics, including background diseases and psychiatric medications, duration of ICU and hospital stay, rates and duration of mechanical ventilation, the characteristics of the weaning process (including the amounts of sedative drugs used before extubation), and ICU complication and extubation rates.

For the full list of variables extracted, see Supplement 2. Broadly, these included patient demographics and medical history, the details of the current admission (for example indication, admission ward), the details of weaning from mechanical ventilation (for example the number of weaning and extubation attempts, planned/unplanned extubation, tracheostomy), and the sedative and hypnotic drugs used during the 48 h before extubation.

### Data sources and measurement

All data were extracted from the SZMC EMRs. These data were either documented in real-time by the treating nursing and medical staff or recorded automatically during admission. Physiological and respiratory parameters, laboratory test results, and ventilation settings were recorded automatically. Drugs (continuous and bolus administration) and their concentrations/doses were documented manually by the nursing staff up to 1st August 2018 and recorded directly from syringe pumps to the EMR after this date. All other data were documented in the EMR by the treating medical staff (doctors and nurses).

Complications that occurred during ICU hospitalization were documented and classified according to ICD-9 diagnosis as they were recorded by the medical staff. For example, secondary infections include central line associated, skin, surgical wound, or gastrointestinal tract infections. Pulmonary complications include hospital-acquired pneumonia, iatrogenic pneumothorax, and lung atelectasis. Sepsis was diagnosed based on the presence of either an ICD-9 code in the discharge summary or a documented septic episode during admission. Patients were followed to hospital discharge or death (whichever occurred first).

### Bias

The file of one patient identified for study inclusion could not be located (1.6%). To calculate the medication dose per body weight, we extracted patient weight from routine nursing admission notes. If it had not been documented at this time, we extracted patient weight from the first assessment performed by the clinical nutritionist after admission or from the weight most recently documented hospital in a previous hospital visit. The files of five patients contained no information regarding weight (2.5%). For these patients we used the average gendered (male/female) weight of the group to which they were allocated (study/control).

### Study size and sample size calculation

We planned a study of independent cases and controls with 2 control(s) per case. Due to the limited number of cases, we used a 1:2 study to control ratio, thereby increasing the power of the study [13, 14]. Prior data indicate that the likelihood of death among ICU patients with no ID approximates 25–30% [15, 16]. Prior data also allows us to assume that the true odds ratio for death in critically ill patients with ID relative to those without ID approximates 2.5 [17, 18]. We therefore calculated we would need to study 59 patients with ID and 118 patients without ID to be able to reject the null hypothesis that this odds ratio equals 1 with probability (power) 0.8. The type I error probability associated with this test of this null hypothesis is 0.05.

### Variable definitions and management of quantitative variables

Patients referred to the hospital from a nursing home or an assisting community/facility were listed as “admitted from institution”. The cause of admission was listed as surgical if the patient arrived to the ICU from a surgical ward or if the patient underwent surgery during ICU admission. A weaning attempt was defined as such if the ventilation method was changed from intermittent mechanical ventilation (in any mode) to continuous positive airway pressure (CPAP) with pressure support (PS).

If the patient underwent more than one intubation, we included weaning and medication data only from the first extubation attempt but documented failed extubations, reintubation, and/or tracheotomies (if performed).

Drug doses were calculated as the overall amount given in 24 h (continuous and bolus doses) divided by the actual duration of administration (for example total 200 ml divided by 10 h of continuous infusion) and were calculated with respect to the patient's weights (in mg/kg/h).

### Statistical methods

Categorical variables are described as frequencies and percentages. Quantitative variables are presented as means with their standard deviations [SDs], as well as median and interquartile ranges [IQRs]. After examining variable distributions, the groups (study/control) were compared using either Chi-square or Fisher’s exact tests for categorical variables and either unpaired Student t-test or Mann–Whitney tests for quantitative variables, dependent on variable distribution. All statistical tests were two-sided and a *p* value ≤ 0.05 was considered statistically significant. Statistical analyses were all performed using SPSS (IBM Corp. Released 2017. IBM SPSS Statistics for Windows, Version 25.0. Armonk, NY: IBM Corp.)

## Results

**Fig. 1:**
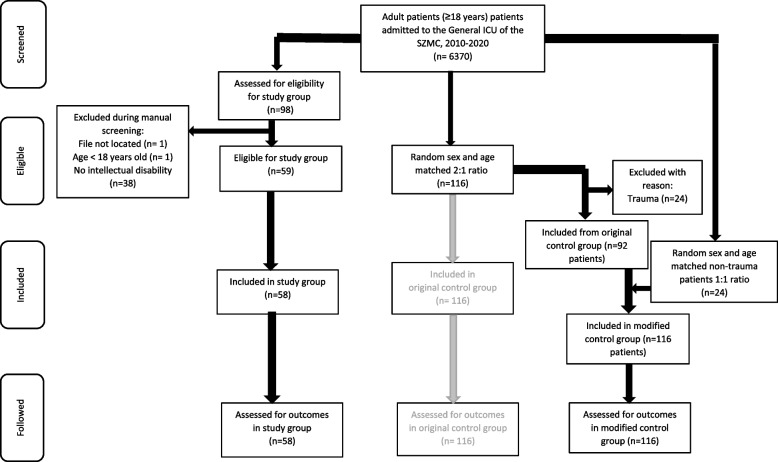
Flowchart of population inclusion/exclusion process

Based on study period and ICD-9 codes, overall 98 patients were identified as potentially eligible for inclusion in the study group. Forty were excluded during manual chart review yielding a final study group comprised of 58 patients (see Fig. 1 for details of the inclusion-exclusion process). The control group included 116 patients with no intellectual disability ho had been admitted to the ICU during the study period and had similar age and sex as the study group patients.

Preliminary review of study and control group characteristics revealed significantly more patients with physical trauma and motor vehicle accidents among controls. We therefore randomly selected 24 substitute control patients to adjust for this difference, thereby creating a modified control group that better matched the baseline characteristics of the study group (referred as “original”; with trauma patients, and “modified”; without trauma patients, Supplement 4a). Ultimately, data were collected on 140 controls and the final dataset was comprised of 198 patients overall. In the main body of the article the data are presented for the modified control group. The data for the original control group are presented as supplementary material.

### Description of the cohort as a whole

The mean age of the cohort as a whole was 39.19 ± 14.02 years (median 35, IQR 28.75–49.25, range 18–70). Overall, 70.2% (*n* = 139) were male and 29.8% (*n* = 59) were female. Their mean APACHE II score was 14.5 ± 8.7 (median 14, IQR 8–19). Additional characteristics are presented in Table 1.

**Table 1:** Baseline characteristics of critically ill patients with intellectual disability versus matched patients without intellectual disability

		Study group with control (***n*** = 174)	Study group (***n*** = 58)	control (***n*** = 116)	***p*** value	95% CI
**General demographics**						
Age (years) Mean±SD [median; IQR; min–max]		39.5 ± 13.9 [39; 28.8–49.3; 18–70]	39.6 ± 13.9 [39.5; 28.8–49.3; 18–70]	39.4 ± 14 [39; 28.3–49.8 ; 18–70]	Matched	
Sex (M/F) % (*n*)		67.2%/32.8% (117/57)	67.2%/32.8% (39/19)	67.2%/32.8% (78/38)	Matched	
Admitted from (home/institution) % (*n*)		78.7/21.3 (137/37)	41.4%/58.6% (24/34)	97.4%/2.6% (113/3)	< 0.001	0.99, 1.04
**Severity of acute disease assessment**						
APACHE II score Mean±SD [median; IQR; min–max]		15.1 ± 8.9 [12.5; 8.8–20; 0–44]	18.5 ± 8.7 [18; 11–23.3; 4–44]	13.4 ± 8.5 [12.5; 7–18; 0–43]	< 0.001	11.6, 14.5
Use of vasopressors % (*n*)		35.1% (61)	51.7% (30)	26.7% (31)	**0.001**	0.02, 0.56
Use of renal replacement therapy % (*n*)		1.2% (2)	1.7% (1)	0.9% (1)	*p* = 0.615	0, 0
**Admission characteristics**						
Surgical/non-surgical admission % (*n*)		44.3%/55.7% (77/97)	37.9%/62.1% (22/36)	47.4%/52.6% (55/61)	0.235	1.35, 1.53
Referring department % (*n*)	Internal i	21.8% (38)	22.4% (13)	21.6% (25)	0.213	2.53, 3.07
	Surgical ii	17.8% (31)	22.4% (13)	15.5% (18)		
	Gynecology	6.9% (12)	3.4% (2)	8.6% (10)		
	Emergency department	39.7% (69)	46.6% (27)	36.2% (42)		
	Neurosurgery	10.9% (19)	5.2% (3)	13.8% (16)		
	Unknown	2.9% (5)	0% (0)	4.3% (5)		
Cardiovascular disease % (*n*)		18.4% (32)	22.4% (13)	16.4% (19)	0.333	0.08, 0.21
Hypertension % (*n*)		16.1% (28)	15.5% (9)	16.4% (19)	0.884	0.09, 0.22
Pulmonary disease % (*n*)		8% (14)	5.2% (3)	9.5% (11)	0.324	0.02, 0.12
Renal disease % (*n*)		6.3% (11)	3.4% (2)	7.8% (9)	0.271	0.03, 0.13
Hematological disease % (*n*) iii		13.2% (23)	20.7% (12)	9.5% (11)	0.040	0.02, 0.12
Malignancy % (*n*)		12.1% (21)	12.1% (7)	12.1% (14)	1	0.06, 0.18
Endocrinological condition (other than diabetes mellitus) % (*n*) iv		16.7% (29)	31.0% (18)	9.5% (11)	< 0.001	0.02, 0.12
Diabetes mellitus % (*n*)		19.0% (33)	17.2% (10)	19.8% (23)	0.682	0.12, 0.26
Obesity % (*n*) v		14.4% (25)	19.0% (11)	12.1% (14)	0.221	0.03, 0.14
Neurological disease % (*n*) vi		24.7% (43)	37.9% (22)	18.1% (21)	0.004	0.09, 0.22
Psychiatric disorders % (*n*) vii		19.0% (33)	25.9% (15)	15.5% (18)	0.101	0.08, 0.21
Chronic infectious disease % (*n*) viii		8.0% (14)	10.3% (6)	6.9% (8)	0.431	0.02, 0.12
Smoking % (*n*)		24.7% (43)	6.9% (4)	33.6% (39)	< 0.001	0.25, 0.42
Other disease % (*n*) ix		35.1% (61)	37.9% (22)	33.6% (39)	0.574	0.23, 0.40

^i^Includes Hematology-Oncology, Otolaryngology (1 patient–without surgical intervention), Cardiac Intensive Care Unit, Cardiology, Neurology

^ii^Includes Otolaryngology/Head and Neck Surgery, Orthopedics, Vascular surgery, Urology

^iii^Includes anemia, polycythemia, thalassemia, coagulation disorders, hematological malignancy

^iv^Includes hyper/hypo-thyroidisim, hyper/hypo-parathyroidism, osteoporosis, Addisson disease, polycystic ovary syndrome, Cushing syndrome, panhypopituitirism

^v^Based on International Clinical Diseases-9 diagnosis code

^vi^Neuromuscular diseases, epilepsy, dementia, stroke, arterio-venous malformation, migranes

^vii^Autism, schizophrenia, schizo-affective disorder, depression, drug and alcohol abuse

^viii^Hepatitis B, hepatitis C, HIV, recurrent infections, Carbapenem Rresistent Klabsiella Pneumonia/Vancomycon Resistent Enterococcus carriers

^ix^Rheumatology disorders, inflammatory bowel disease, genetic disorders, underwent major surgeries, allergies, gastrointestinal conditions, immune deficiencies, pregnancy

Statistically significant findings (*p* ≤ 0.05) are highlighted. The 95% CI refers to the difference between proportions

### Description of study and control patients

The study group was comprised of patients with general mental retardation (70.7%, *n* = 41), Down syndrome (27.6%, *n* = 16), and panhypopituitarism (1.7% *n* = 1). The study and control groups were well matched in age and sex as planned. However, only 41.4% of study patients were admitted from home compared 97.4% in the control group (*p* < 0.001) (Table 1).

### Severity of acute disease

The mean APACHE II score of study group was 18.5 ± 8.7 (median 18, IQR 11–23.25, range 4–44) whereas the control group had a mean APACHE II score of 13.4 ± 8.5 (median 12.5, IQR 7–18, range 0–43, *p* < 0.001). Also, the proportion of patients receiving vasopressors was higher among study patients than among control patients (51.7% vs. 26.7%, *p* = 0.001).

### Primary outcome measure: ICU and in-hospital mortality

The ICU mortality rate was 5.2% (*n* = 3) among study patients versus 4.3% (*n* = 5) among control patients (*p* = 0.0798, 95%CI − 0.08, 0.23, OR 1.2566). The in-hospital mortality rate was 19% (*n* = 11) among study patients and 9.5% (*n* = 11) among control patients (*p* = 0.076, 95%CI − 0.07, 0.35, OR 2.2340) (Table 1).

### Secondary outcome measures: background diseases and psychiatric/neurological medications

Study patients had more comorbidities than control patients, including hematological (20.7% vs. 9.5%, *p* = 0.04), endocrinological (excluding diabetes) (31% vs. 9.5%, *p* < 0.001), and neurological diseases (37.9% vs. 18.1%, *p* = 0.004). Conversely, the proportion of smokers was lower among study patients (6.9% vs. 33.6% control, *p* < 0.001) (Table 1).

The prevalence of psychiatric medication use before admission was higher among study patients vs. control patients (53.4% vs. 14.7% respectively, *p* < 0.001). Specifically, study patients were treated more with anti-depressants, typical anti-psychotics, atypical anti-psychotics, benzodiazepines, anti-convulsive drugs, and anti-cholinergic medications (Table 2).

**Table 2:** Pre-admission treatment with psychiatric drugs among among critically ill patients with intellectual disability versus matched patients without intellectual disability

	Study group with control (***n*** = 174) % (***n***)	Study group (***n*** = 58) % (***n***)	Control (***n*** = 116) % (***n***)	***p*** value	95% CI
Yes	27.6% (48)	53.4% (31)	14.7% (17)	**< 0.001**	0.06, 0.18
Antidepressants	5.7% (10)	12.1% (7)	2.6% (3)	**0.017**	0, 0.06
Typical antipsychotics	4.6% (8)	13.8% (11)	0% (0)	**< 0.001**	0, 0
Atypical antipsychotics	10.3% (18)	20.7% (12)	5.2% (6)	**0.002**	0.01, 0.09
Benzodiazepines	10.3% (18)	20.7% (12)	5.2% (6)	**0.002**	0.01, 0.09
Mood stabilizers	0.6% (1)	0% (0)	0.9% (1)	1	− 0.01, 0.03
Barbiturates	1.1% (2)	3.4% (2)	0% (0)	0.110	0, 0
Anti-convulsants	15.5% (27)	25.9% (15)	10.3% (12)	**0.008**	0.02, 0.12
Anticholinergics	3.4% (6)	8.6% (5)	0.9% (1)	**0.008**	− 0.01, 0.03
Stimulants/psychoactives	0.6% (1)	1.7% (1)	0% (0)	0.333	0, 0

Statistically significant findings (*p* ≤ 0.05) are highlighted. The 95% CI refers to the difference between proportions

### Rate and duration of mechanical ventilation and weaning

#### Rate of mechanical ventilation

The proportion of patients undergoing intubation and mechanical ventilation was higher among study patients than among control patients (86.2% [*n* = 50], vs. 37.9% [*n* = 44], *p* < 0.001).

#### Duration of mechanical ventilation and weaning

The overall duration of mechanical ventilation (comprised of the time from intubation to first weaning attempt and the time from initiation of the first weaning attempt to extubation) was longer in study patients than in control patients; study patients 7.3 ± 6.2 days (median 5, IQR 2–11), versus control patients 4.1 ± 2.8 days (median 4, IQR 2–5) (*p* = 0.005, 95% CI 1.05, 5.45).

The time from intubation to first weaning attempt was longer in study patients than in control patients (4.8 ± 5.0 days [median 1, IQR 1–7.5], versus 2.7 ± 2.8 [median 1, IQR 1–4], *p* = 0.02, 95% CI 0.34, 3.80). Also, the time from initiation of the first weaning attempt to successful extubation was longer (1.5 ± 2.5 days [median 1, IQR 0–2], vs 0.48 ± 1.40 [median 1, IQR 0–1], *p* = 0.007, 95% CI 0.30, 1.78).

Finally, tracheostomy rates were significantly higher in study patients compared to control patients (20.7% [*n* = 12], vs. 2.6% [*n* = 3], *p* < 0.001) (Table 3).

**Table 3:** Intubation characteristics and the weaning process among critically ill patients with intellectual disability versus matched patients without intellectual disability

	Study group (***n*** = 58)	Modified controls (***n*** = 116)	***p*** value	95% CI
Proportion of patients intubated (within the full cohort) % (*n*)	86.2% (50/58)	37.9% (44/116)	**< 0.001**	0.77, 0.95
Time from intubation to first weaning attempt (days) Mean±SD [median; IQR; min–max]	4.8 ± 5.04 [1; 1–7.5; 0–23]	2.7 ± 2.8 [1; 1–4; 0–11]	**0.020**	0.34, 3.8
Time from first weaning attempt to extubation (days) Mean±SD [median; IQR; min–max]	1.5 ± 2.5 [1; 0–2; 0–14]	0.48 ± 1.4 [1; 0–1; 0–14]	**0.007**	0.3, 1.78
Intubation duration (days) Mean±SD [median; IQR; min–max]	7.3 ± 6.2 [5; 2–11; 1–24]	4.06 ± 2.7 [4; 2–5; 1–12]	**0.005**	1.05, 5.45
Number of extubation attempts Mean ±SD [median; IQR; min–max]	1.1 ± 0.6 [3; 1–1; 0–3]	0.9 ± 0.4 [3; 1–1; 0–2]	0.093	− 0.03, 0.41
Number of weaning attempts Mean±SD [median; IQR; min–max]	1.19 ± 0.890 [3; 1–2; 0–4]	0.88 ± 0.498 [3; 1–1; 0–2]	0.055	− 0.007, 0.62
Number of self extubations Mean ±SD [median; IQR; min–max]	0.05±0.221 [0; 0-1; 0-1]	0.06±0.233 [0; 0-1; 0-1]	0.7801	-0.01, 0.12
Proportion of ultimately successful extubations % (*n*)	61.2% (30)	75.0% (33)	0.156	0.65, 0.9
Reintubation within 24 h from extubation % (*n*)	5.2% (3)	3.4% (4)	0.592	0, 0.16
Tracheostomy (all patients) % (*n*)	20.7% (12)	2.6% (3)	**< 0.001**	− 0.1, 0.13
Tracheostomy (intubated patients) % (*n*)	24% (12)	6.1% (3)	**0.013**	− 0.1, 0.13

The data on extubation of 1 patient was missing (transferred to another hospital while intubated)

Statistically significant differences (*p* < 0.05) are highlighted. The 95% CI refers to the difference between proportions

### Sedative drugs and doses used prior to extubation

Propofol and remifentanil were the drugs most commonly used in the 48 h prior to extubation in both study and control patients. While both propofol and benzodiazepines were used for a significantly greater proportion of patients in the control group than in the study group before extubation, (56%% vs. 77.6%, *p* = 0.023 and 6% vs. 24.5% respectively, *p* = 0.01) (Tables 4 and 5), the overall dose of propofol given in the 48 h prior to extubation was lower in study patients vs. controls, with 1.48 mg/kg/h vs. 2.06 mg/kg/h (*p* = 0.034) in the 0–24 h, and 1.38 mg/kg/h vs. 2.14 mg/kg/h (*p* = 0.009) in the 24–48 h before extubation. No other differences were found when comparing additional sedatives (Table 4).

**Table 4:** Medications given 48 h before extubation among critically ill patients with intellectual disability versus matched patients without intellectual disability

	Study group (***n*** = 58)	Modified controls (***n*** = 116)	***p*** value vs. modified controls	95% CI
Total number of drugs given within 0–48 h prior to extubation. Mean ±SD [median; IQR; min–max]	2.8 ± 1.1 [12; 2–4; 1–6]	3.2 ± 1.5 [12; 2–4; 1–6]	0.192	− 0.06, 0.2
Propofol % (*n*)	56% (28)	77.6% (38)	**0.023**	0.02, 0.56
Midazolam % (*n*)	10% (5)	22.4% (11)	0.092	− 0.06, 0.35
Dexmedetomidine % (*n*)	8% (4)	6.1% (3)	1	0, 0
Morphine % (*n*)	8% (4)	20.4% (10)	0.076	− 0.07–0.35
Fentanyl % (*n*)	12% (6)	20.4% (10)	0.256	− 0.07, 0.35
Remifentanil % (*n*)	40% (20)	44.9% (22)	0.622	− 0.07, 0.35
Ketamine % (*n*)	0% (0)	1% (2)	0.495	0, 0
Methadone % (*n*)	14% (7)	22.4% (11)	0.276	0, 0
Seroquel % (*n*)	8% (4)	18.4% (9)	0.127	0, 0
Clonex % (*n*)	6% (3)	24.5% (12)	**0.010**	0, 0
Halidaol % (*n*)	6% (3)	16.3% (8)	0.102	0, 0
Clozapine % (*n*)	4% (2)	0% (0)	0.495	0.08, 0.22

Statistically significant findings (*p* ≤ 0.05) are highlighted. The 95% CI refers to the difference between proportions

**Table 5:** Total dose of drugs given in the 48 h before extubation among critically ill patients with intellectual disability versus matched patients without intellectual disability

	Study group (***n*** = 58)	Modified controls (***n*** = 116)	***p*** value	95% CI
**IV continuous drugs mg/kg/h**				
Propofol 0–24 h prior to extubation, Mean±SD [median; IQR; min–max]	1.5 ± 1 [1.5; 0.6–1.9; 0.3–3.3]	2.1 ± 1 [1.9; 1.4–2.3; 0.4–6.2]	**0.034**	1.72, 2.4
Propofol 24–48 h prior to extubation, Mean±SD [median; IQR; min–max]	1.4± 0.6 [1.32; 0.8–1.9; 0.4–2.4]	2.1 ± 1.1 [2.1; 1.2–2.5; 0.4–4.9]	**0.009**	1.7, 2.6
Midazolam 0–24 h prior to extubation, Mean ±SD [median; IQR; min–max]	None given	0.12 ± 0.1 [0.09; 0.04–0.2; 0.03–0.33]	0.889	0.04-0.21
Midazolam 24–48 h prior to extubation, Mean±SD [median; IQR; min–max]	None given	0.08 ± 0.07 [0.05; 0.03 − 0.1; 0.02–0.2]	0.571	0.005, 0.15
Morphine 24–48 h prior to extubation, Mean±SD [median; IQR; min–max]	None given	None given		
Fentanyl 0–24 h prior to extubation, Mean ±SD [median; IQR; min–max]	0.02 ± 0.04 [0.001; 0.0007–0.05; 0–0.1]	0.02 ± 0.28 [0.002; 0.0008–0.003; 0–1.05]	0.672	-0.05, 0.26
Fentanyl 24–48 h prior to extubation, Mean ±SD [median; IQR; min–max]	0.06 ± 0.1 [0.002; 0.001–0.003; 0–0.2]	0.003 ± 0.001 [0.003; 0.001− 0.003; 0–0.1]	1	0.002, 0.004
Remifentanil 0–24 h prior to extubation, Mean±SD [median; IQR; min–max]	0.01± 0.02 [0.006; 0.003 − 0.009; 0–0.08]	0.01 ± 0.01 [0.008; 0.004–0.01; 0–0.06]	0.331	0.005, 0.015
Remifentanil 24–48 h prior to extubation, Mean ±SD [median; IQR; min–max]	0.006 ± 0.003 [0.007;0.005 − 0.001; 0–0.01]	0.008 ± 0.003 [0.007; 0.005–0.01; 0–0.01]	0.516	0.006,0.009
Dexmedetomidine 24–48 h prior to extubation, Mean ±SD [median; IQR; min–max]	None given	0.4 ± 0.13 [0.4; 0.2–0.4; 0.3–0.5]	0.5	− 0.95, 1.64
Dexmedetomidine 0–24 h prior to extubation, Mean±SD [median; IQR; min–max]	0.6 ± 0.3 [0.6; 0.35–0.9; 0.3–1]	0.6 ± 0.1 [0.6; 0.5–0.7; 0.5–0.7]	1	0.49, 0.69
**Drugs given by bolus, mg**				
Methadone 0–24 h prior to extubation, Mean±SD [median; IQR; min–max]	44 ± 36.5 [30; 15–80; 10–100]	48.3 ± 43.4 [30; 12.5–50; 5–120]	0.898	14.93, 81.72
Methadone 24–48 h prior to extubation, Mean ±SD [median; IQR; min–max]	48 ± 35.8 [60; 10–80; 10–85]	70 ± 36.5 [60; 32.5–67.5; 30–120]	0.530	36.22, 103.77
Clonex 0–24 h prior to extubation, Mean ±SD [median; IQR; min–max]	0.91 ± 0.63 [1; 0.25–1; 0.25–1.5]	1.9 ± 1.7 [1; 0.75–1.9; 0.5–6]	0.209	0.66, 3.27
Clonex 24–48 h prior to extubation, Mean ±SD [median; IQR; min–max]	None given	2.9 ± 1.7 [2.5; 1.5–4; 1.5–6]	0.286	1.11, 4.71
Quetiapine 0–24 h prior to extubation, Mean ±SD [median; IQR; min–max]	187.5 ± 176.2 [112.5; 81.25–368.75; 75–450]	72.7 ± 36.1 [75 ; 37.5–93.8; 25–150]	0.106	48.44, 97.01
Quetiapine 24–48 h prior to extubation, Mean ±SD [median; IQR; min–max]	133.8 ± 86.9 [112.5; 63.75–225; 60–250]	58.9 ± 47.2 [50; 15.6–75; 12.5–150]	0.190	15.3, 102.55
Clozapine 0–24 h prior to extubation, Mean ±SD [median; IQR; min–max]	500 ± 141.4 [500; 400–600; 400–600]	None given		
Clozapine 24–48 h prior to extubation, Mean±SD [median; IQR; min–max]	500± 141.4 [500; 400–600; 400–600]	None given		
Halidol 0–24 h prior to extubation, Mean ±SD [median; IQR; min–max]	3.5 ± 2.1 [3.5, 2–3.5; 2–5]	20 ± 21.7 [10; 5–37.5; 5–60]	0.143	− 2.75, 42.75

Statistically significant findings (*p* ≤ 0.05) are highlighted. The 95% CI refers to the difference between proportions

### ICU complications

Study patients had more ICU complications (34.5% vs. 8.6%, *p* < 0.001); specifically secondary infections (29.3% vs. 2.6%, *p* < 0.001), pulmonary complications (8.6% vs. 1.7%, *p* = 0.029) and sepsis (6.9% vs. 0%, *p* = 0.012).

### Lengths of stay and readmission rates

Study patients had longer ICU LOSs (mean 10.95 ± 11.4 days, median 5.5, IQR 3–16) than control patients (mean 4.9 ± 3.7 days, median 6, IQR 2–7) (*p* = 0.001, 95% CI 2.24, 8.41). Study patients also had longer hospital LOSs (mean 30.7 ± 40.3 days, median 20, IQR 8.75–36) than control patients (mean 16.4 ± 16.9 days, median 24, IQR 6.25–19.75) (*p* = 0.032, 95% CI 1.07, 23.24).

Study patients had a lower rate of readmission to the ICU during the same hospital stay compared to control patients (6.9% vs. 8.6% respectively, *p* = 0.036). Conversely, study patients had a higher rate of second hospitalization with ICU admission within the study period compared to control patients (0.38 ± 0.721 vs. 0.16 ± 0.486 respectively, *p* = 0.024) (Table 6).

**Table 6:** Admission characteristics and mortality rates among critically ill patients with intellectual disability versus matched patients without intellectual disability

	Study group (***n*** = 58)	Control (***n*** = 116)	***p*** value	95% CI
Overall hospital admission duration (days) Mean ±SD [median; IQR; min–max]	30.7 ± 40.3 [20; 8.8–36 ; 0–230]	16.4 ± 16.9 [24; 6.3– 19.8; 2–90]	**0.032**	1.07, 23.2
ICU admission duration (days) Mean±SD [median; IQR; min–max]	11 ± 11.4 [5.5; 3–16 ; 0–60]	4.9 ± 3.7 [6; 2–7; 1–18]	**0.001**	2.24, 8.41
Second admissions to the hospital with ICU admission Mean ±SD [median; IQR; min–max]	0.4 ± 0.7 [1; 0–1; 0–3]	0.2 ± 0.5 [1; 0–0; 0–3]	**0.024**	0.01, 0.43
Readmissions to the ICU within the same hospital admission % (*n*)	6.9% (4)	8.6% (10)	**0.036**	0.54, 1.03
Mortality in the ICU % (*n*)	5.2% (3)	4.3% (5)	0.798	− 0.08, 0.23
Overall mortality (in the ICU and outside of the ICU) % (*n*)	19% (11)	9.5% (11)	0.076	− 0.07–0.35
Complication during ICU hospitalization % (*n*)	34.5% (20)	8.6% (10)	**< 0.001**	0.07, 0.64
cardiac complication during ICU hospitalization % (*n*)	3.4% (2)	0.9% (1)	0.217	− 0.08, 0.23
Infection complication during ICU hospitalization % (*n*)	29.3% (17)	2.6% (3)	**< 0.001**	− 0.07, 0.35
pulmonary complication during ICU hospitalization % (*n*)	8.6% (5)	1.7% (2)	**0.029**	− 0.07, 0.35
renal complication during ICU hospitalization % (*n*)	5.2% (3)	1.7% (2)	0.335	0, 0
neurological complication during ICU hospitalization % (*n*)	5.2% (3)	2.6% (3)	0.402	− 0.08, 0.23
sepsis complication during ICU hospitalization % (*n*)	6.9% (4)	0% (0)	**0.012**	0, 0
Pressure ulcer complication during ICU hospitalization % (*n*)	0% (0)	0% (0)	Irrelevant	0, 0
Pulmonary embolism complication during ICU hospitalization % (*n*)	0% (0)	0% (0)	Irrelevant	0, 0
DVT complication during ICU hospitalization % (*n*)	1.7% (1)	0% (0)	0.333	0, 0

Rates were calculated from the entire group (*n* = 174) with no missing data. Statistically significant differences (*p* < 0.005) are highlighted. The 95% CI refers to the difference between proportions

## Discussion

Although the current study identified several characteristics related to ICU admission that seem unique to patients with intellectual disability, no difference in mortality was found. These patients had more background diseases and received more psychiatric/neurological medications before admission than their random age- and sex-matched controls. They presented with significantly higher APACHE II scores and had a higher rate of vasopressor use. These patients were more likely to undergo mechanical ventilation than random age- and sex-matched controls, had longer durations of intubation, underwent more weaning attempts despite less use of sedative/analgesic medications during the process of weaning, and underwent more tracheostomies. These patients also had more complications during admission and, unsurprisingly, they had longer ICU and hospital stays. Finally, although their readmission rates were lower and their survival outcomes were no different than those of random age- and sex-matched controls without intellectual disability, they more often had a repeated hospitalization with ICU admission.

The findings of our study are reminiscent of some other studies that describe no difference in mortality rates in patients with and without ID [18], despite higher intubation rates [19] more frequent vasopressor use [18, 19], and more sepsis complications in ID patients [7, 12]. Other studies showed significantly higher ICU admission rates [6] and more prolonged ICU [4, 6] and hospital admissions [4]. Taken together, the findings of our study and their similarity to other studies, suggest patients with intellectual disability may survive admission at a higher cost in terms of quality of life and level of post-discharge independent functioning. Alternatively, our sample size may have been too small to identify differences in survival or this may be a phenomenon observed specifically in our medical center.

Despite our small number of cases our study has several advantages. Contrary to many other clinical and epidemiological studies on this population, we compared ID patients to randomly selected age- and sex-matched controls [18–20]. While the size of our dataset did not enable propensity scoring, this partial matching eliminates at least some potential bias and the remaining between-group differences are now highlighted, enabling adjustment in future studies. In addition, manual data extraction ensured a low rate of missing data. It also enabled focus on details of the medications administered and the weaning process that are not easily accessible in most hospital databases.

Our findings indicate several potential causes of worse outcomes in adult ID patients. These patients seem to have more background diseases, higher APACHE II scores and increased rates of vasopressor use and are therefore probably in a worse clinical condition at the time of ICU admission, than their age- and sex-matched counterparts. Higher APACHE scores and vasopressor requirements may also indicate the presence of lower physiological reserves in this population. Finally, these findings may also be indirect evidence for late diagnosis and treatment due to difficulties encountered in understanding, communicating, or identifying symptoms in ID patients [21].

One would also expect that ID patients would require heavier sedation, due to a combination of preadmission habituation to drugs and poor cooperation with the weaning process. In this study, this did not seem to be the case, as these patients underwent much longer durations of mechanical ventilation despite the use of less medication. Possible explanations for this finding include the higher burden of prior comorbidities, previous sedentary lifestyle with prior muscle wasting, and/or greater sensitivity to sedative drug effects [22, 23].

Our study has several limitations. Given the small number of cases and the many differences we identified between adults with and without ID despite our best efforts to match the two populations, we provide only unadjusted data for the main outcome. Even without adjustment our study is probably underpowered for the main outcome; despite covering a full 10 years of admissions we identified only 58 patients for inclusion in the study group. Moreover, this study was conducted in a single medical center. This not only limited the number of candidates for study inclusion but could also affect generalizability. While prior papers on adult populations also found no difference in mortality when comparing those with ID to those without [4, 18], these studies also included a small number of medical centers or selected populations. Furthermore, based on the mortality rates observed in our study, we calculated that a sample size of more than 6500 cases and 13,000 controls would be required to identify a difference in mortality.

In conclusion, adults with intellectual disability that are admitted to the ICU differ significantly from their age- and sex-matched counterparts. These patients seem sicker at the time of admission, require more supportive treatment and their process of weaning from mechanical ventilation is more complex. These findings are highly supportive of the need for individualized patient care in the ICU. Our findings require validation in multicenter data with larger sample sizes.

## Supplementary Information

**Additional file 1 Supplement 1.** Link to the free Python random module: https://miniwebtool.com/random-picker. **Supplement 2.** Table listing the variables extracted for the purpose of the current study. **Supplement 3.** Search terms used. **Supplement 4a.** Baseline characteristics of critically ill patients with intellectual disability versus matched patients without intellectual disability. **Supplement 4b.** Pre-admission treatment with psychiatric drugs among among critically ill patients with intellectual disability versus matched patients without intellectual disability. **Supplement 5.** intubation characteristics and the weaning process among critically ill patients with intellectual disability versus matched patients without intellectual disability. **Supplement 6a.** Medications given 48 hours before extubation among critically ill patients with intellectual disability versus matched patients without intellectual disability. **Supplement 6b.** Total dose of drugs given in the 48 hours before extubation among critically ill patients with intellectual disability versus matched patients without intellectual disability. **Supplement 7.** Admission characteristics and mortality rates among critically ill patients with intellectual disability versus matched patients without intellectual disability. **Supplement 8.** Main diagnosis at the time of intensive care admission according to the International classification of Diseases-9, among critically ill patients with intellectual disability versus matched patients without intellectual disability.

## Data Availability

The datasets during and/or analyzed during the current study available from the corresponding author on reasonable request.

## References

[1] Lunsky Y, De Oliveira C, Wilton A, Wodchis W (2019). High health care costs among adults with intellectual and developmental disabilities: a population-based study. J Intellect Disabil Res..

[2] Amor-Salamanca A, Menchon JM (2018). Rate and characteristics of urgent hospitalisation in persons with profound intellectual disabilities compared with general population. J Intellect Disabil Res..

[3] Iwase S, Bérubé NG, Zhou Z, Kasri NN, Battaglioli E, Scandaglia M, Barco A (2017). Epigenetic etiology of intellectual disability. J Neurosci..

[4] Tenenbaum A, Chavkin M, Wexler ID, Korem M, Merrick J (2012). Morbidity and hospitalizations of adults with Down syndrome. Res Dev Disabil..

[5] Foreman KJ, Abbasoglu OA (2015). Global, regional, and national disability-adjusted life years (DALYs) for 306 diseases and injuries and healthy life expectancy (HALE) for 188 countries, 1990-2013: quantifying the epidemiological transition. Lancet..

[6] Ailey SH, Johnson T, Fogg L, Friese TR (2014). Hospitalizations of adults with intellectual disability in academic medical centers. Intellect Dev Disabil..

[7] Lin JA, Liao CC, Chang CC, Chang H, Chen TL (2011). Postoperative adverse outcomes in intellectually disabled surgical patients: a nationwide population-based study. PLoS One.

[8] Brown HK, Saha S, Chan TCY (2022). Outcomes in patients with and without disability admitted to hospital with COVID-19: a retrospective cohort study. CMAJ..

[9] Florio T, Trollor J (2015). Mortality among a cohort of persons with an intellectual disability in New South Wales, Australia. J Appl Res Intellect Disabil.

[10] Glover G, Williams R, Oyinlola J (2020). An observational cohort study of numbers and causes of preventable general hospital admissions in people with and without intellectual disabilities in England. J Intellect Disabil Res..

[11] Russel T, Hahn J, Hayward K (2017). Psychiatric services for individuals with intellectual and developmental disabilities: medication management. J Mental Health Res Intellectual Disabil.

[12] von Elm E, Altman DG, Egger M, Pocock SJ, Gøtzsche PC, Vandenbroucke JP, STROBE Initiative (2008). The Strengthening the Reporting of Observational Studies in Epidemiology (STROBE) statement: guidelines for reporting observational studies. J Clin Epidemiol..

[13] Hennessy S, Bilker WB, Berlin JA, Strom BL (1999). Factors influencing the optimal control-to-case ratio in matched case-control studies. Am J Epidemiol..

[14] Kang M-S (2009). The effect of increasing control-to-case ratio on statistical power in a simulated case-control SNP association study. Genomics Inform.

[15] Weigl W, Adamski J, Goryński P, Kański A, Hultström M (2018). ICU mortality and variables associated with ICU survival in Poland: a nationwide database study. Eur J Anaesthesiol..

[16] Capuzzo M, Volta C, Tassinati T, Moreno R, Valentin A, Guidet B, Iapichino G, Martin C, Perneger T, Combescure C, Poncet A, Rhodes A, Working group on health economics of the european society of intensive care medicine (2014). Hospital mortality of adults admitted to Intensive Care Units in hospitals with and without Intermediate Care Units: a multicentre European cohort study. Crit Care..

[17] Baraona F, Gurvitz M, Landzberg MJ, Opotowsky AR (2013). Hospitalizations and mortality in the United States for adults with Down syndrome and congenital heart disease. Am J Cardiol..

[18] Tibby SM, Durward A, Goh CT, Thorburn K, Morris K, Broadhead M, Peters MJ (2012). Clinical course and outcome for critically ill children with Down syndrome: a retrospective cohort study. Intensive Care Med..

[19] Joffre C, Lesage F, Bustarret O, Hubert P, Oualha M (2016). Children with Down syndrome: clinical course and mortality-associated factors in a French medical paediatric intensive care unit. J Paediatr Child Health..

[20] Strauss D, Eyman RK, Grossman HJ (1996). Predictors of mortality in children with severe mental retardation: the effect of placement. Am J Public Health..

[21] Amor-Salamanca A, Menchon JM (2017). Pain underreporting associated with profound intellectual disability in emergency departments. J Intellect Disabil Res..

[22] Chaudhary K, Bagharwal P, Wadhawan S (2017). Anesthesia for intellectually disabled. J Anaesthesiol Clin Pharmacol.

[23] Costello A, Hudson E, Morrissey S, Sharma D, Kelly D, Doody O (2022). Management of psychotropic medications in adults with intellectual disability: a scoping review. Ann Med.

