# Supplementary material for: Intellectually disabled patients’ intensive care admission characteristics, weaning from mechanical ventilation, and sedative drug use: a single-center retrospective case-control study
Source: J Anesth Analg Crit Care. 2022 Dec 21;2:52. doi: 10.1186/s44158-022-00081-4 (PMC10246319; doi:10.1186/s44158-022-00081-4)
Supplement: Supplementary file 1 — Additional file 1 Supplement 1. Link to the free Python random module: https://miniwebtool.com/random-picker. Supplement 2. Table listing the variables extracted for the purpose of the current study. Supplement 3. Search terms used. Supplement 4a. Baseline characteristics of critically ill patients with intellectual disability versus matched patients without intellectual disability. Supplement 4b. Pre-admission treatment with psychiatric drugs among among critically ill patients with intellectual disability versus matched patients without intellectual disability. Supplement 5. intubation characteristics and the weaning process among critically ill patients with intellectual disability versus matched patients without intellectual disability. Supplement 6a. Medications given 48 hours before extubation among critically ill patients with intellectual disability versus matched patients without intellectual disability. Supplement 6b. Total dose of drugs given in the 48 hours before extubation among critically ill patients with intellectual disability versus matched patients without intellectual disability. Supplement 7. Admission characteristics and mortality rates among critically ill patients with intellectual disability versus matched patients without intellectual disability. Supplement 8. Main diagnosis at the time of intensive care admission according to the International classification of Diseases-9, among critically ill patients with intellectual disability versus matched patients without intellectual disability. [file 44158_2022_81_MOESM1_ESM.docx]

**Supplementary materials**

**Supplement 1:** Link to the free Python random module:

<https://miniwebtool.com/random-picker>

**Supplement 2:** Table listing the variables extracted for the purpose of the current study

| **Demographic Data** | **Hospitalization Data** | **Mechanical Ventilation Data** | **Drug Data** |
| --- | --- | --- | --- |
| - Age - Sex - Weight - Referred to the hospital from home or institution - Background disease:   - Cardiovascular   - Hypertension   - Pulmonary   - Renal   - Malignancy   - Hematological   - Endocrinological (other than diabetes mellitus)   - Diabetes mellitus   - Obesity   - Smoking   - Chronic infectious   - Other - Psychiatric treatment   - Anti-depressants   - Typical antipsychotic   - Atypical antipsychotic   - Benzodiazepines   - Mood stabilizer   - Barbiturates   - Anti-convulsive   - Anti-cholinergic   - stimulant | - Surgical/non-surgical admission - Referring department (internal, surgical, gynecology, ER or neurosurgery) - APACHE II score on admission. - Hospital admission dates - ICU admission dates - Readmissions to the ICU - Mortality in the ICU - Mortality in the hospital following discharge from the ICU - Complications:   - Cardiovascular   - Pulmonary   - Renal   - Infectious   - Neurological   - Sepsis   - Pressure ulcers   - Deep vein thrombosis   - Pulmonary embolism   - Life support use   - Vasopressors   - Renal replacement therapy | - Intubation - Intubation date - Number of total intubations - Number of weaning attempts - Date of first weaning attempt - Number of extubation attempts - Date of first extubation attempt - Number of self0extubations - Mechanical ventilation duration - Re-intubation within 24 hours of extubation - Tracheostomy - Air support given following extubation - Oxygen support given following extubation | - Number of sedative drugs given within 48 hours prior to extubation - Average dose given to the patient within 0-24 and 24-48 hours prior to extubation of the following drugs:   - Propofol   - Midazolam   - Morphine   - Dexmedetomidine   - Fentanyl   - Remifentanil   - Clonex   - Halidol   - Methadone   - Ketamine   - Clozapine   - Seroquel - Doses of sedative drugs given by bolus - Sedative drugs given following extubation |

**Supplement 3**

**Search terms used**

- ICU AND Intellectual Disability
- ICU AND Mental Retardation
- ICU AND Down Syndrome
- Mechanical ventilation AND Mental Retardation
- Mechanical ventilation AND Intellectual Disability
- Mechanical ventilation AND Down Syndrome
- Extubation AND Mental Retardation
- Extubation AND Intellectual Disability

Supplement 4a: Baseline characteristics of critically ill patients with intellectual disability versus matched patients without intellectual disability

|  | | **Study group with original controls**  **(n=174)** | **Study group with modified controls**  **(n=174)** | **Study group**  **(n=58)** | **Original Controls**  **(n=116)** | **Modified controls  (n=116)** | **p-value** vs. original controls | **95% CI** | **p-value** vs. modified controls | **95% CI** |
| --- | --- | --- | --- | --- | --- | --- | --- | --- | --- | --- |
| **General demographics** | | | | | | | | | | |
| Age (Years)  Mean$\pm SD$  [median; IQR; min-max] | | 39.4±14 [39; 28.8-49.3; 18-70] | 39.5±13.9  [39;28.8  -49.3; 18-70] | 39.6±13.9 [39.5;28.8-49.3; 18-70] | 39.3±14.1 [39; 28.3-49.8; 18-70] | 39.4±14  [39; 28.3-49.8 ; 18-70] | Matched | | Matched | |
| Sex (M/F)  % (n) | | 67.2%/  32.8%  (117/  57) | 67.2%/  32.8%  (117/  57) | 67.2%/  32.8%  (39/19) | 67.2%/  32.8%  (78/38) | 67.2%/  32.8%  (78/38) | Matched | | Matched | |
| Admitted from (Home/Institution)  % (n) | | 79.3%/20.7% (138/36) | 78.7/  21.3  (137/37) | 41.4%  /58.6%  (24/34) | 98.3%/  1.7%  (114/2) | 97.4%/  2.6%  (113/3) | <0.001 | 0.99, 1.04 | <0.001 | 0.99, 1.04 |
| **Severity of acute disease assessment** | | | | | | | | | | |
| APACHE II score  Mean$\pm SD$  [median; IQR; min-max] | | 14.9±8.5  [18; 9-19; 0-44] | 15.1±8.9  [12.5; 8.8-20; 0-44] | 18.5±8.7  [18; 11-23.3; 4-44] | 13±7.8  [12.5; 7.3- 17; 0-43] | 13.4±8.5 [12.5; 7-18; 0-43] | <0.001 | 11.6, 14.5 | <0.001 | 11.6, 14.5 |
| Use of vasopressors % (n) | | 34.5%  (60) | 35.1%  (61) | 51.7%  (30) | 25.9%  (30) | 26.7%  (31) | **0.001** | 0.02, 0.56 | **0.001** | 0.02, 0.56 |
| Use of renal replacement therapy  % (n) | | 1.2% (2) | 1.2% (2) | 1.7%  (1) | 0.9%  (1) | 0.9%  (1) | p=0.615 | 0, 0 | p=0.615 | 0, 0 |
| **Admission characteristics** | | | | | | | | | | |
| Surgical/Non-surgical admission  % (n) | | 50%/50% (87/87) | 44.3%/  55.7%  (77/97) | 37.9%/  62.1%  (22/36) | 56.0%/  44.0%  (51/116) | 47.4%/  52.6% (55/61) | 0.024 | 1.35, 1.53 | 0.235 | 1.35, 1.53 |
| Referring department  % (n) | Internal i | 19.5% (34) | 21.8% (38) | 22.4%  (13) | 18.1%  (21) | 21.6%  (25) | 0.303 | 2.53, 3.07 | 0.213 | 2.53, 3.07 |
|  | Surgical ii | 23.0%  (40) | 17.8% (31) | 22.4%  (13) | 23.3%  (27) | 15.5%  (18) |  |  |  |  |
|  | Gynecology | 6.3%  (11) | 6.9% (12) | 3.4%  (2) | 7.8%  (9) | 8.6%  (10) |  |  |  |  |
|  | Emergency department | 39.7% (69) | 39.7% (69) | 46.6%  (27) | 36.2%  (42) | 36.2%  (42) |  |  |  |  |
|  | Neurosurgery | 8.0% (14) | 10.9% (19) | 5.2%  (3) | 9.5%  (11) | 13.8%  (16) |  |  |  |  |
|  | Unknown | 3.4% (6) | 2.9% (5) | 0%  (0) | 5.2%  (6) | 4.3%  (5) |  |  |  |  |
| **Background diseases** | | | | | | | | | | |
| Cardiovascular disease  % (n) | | 17.2% (30) | 18.4% (32) | 22.4%  (13) | 14.7%  (17) | 16.4%  (19) | 0.202 | 0.09, 0.21 | 0.333 | 0.08, 0.21 |
| Hypertension  % (n) | | 15.5% (27) | 16.1% (28) | 15.5%  (9) | 15.5%  (18) | 16.4%  (19) | 1 | 0.06, 0.22 | 0.884 | 0.09, 0.22 |
| Pulmonary disease  % (n) | | 6.3% (11) | 8% (14) | 5.2%  (3) | 6.9%  (8) | 9.5%  (11) | 0.660 | 0.02, 0.12 | 0.324 | 0.02, 0.12 |
| Renal disease  % (n) | | 6.3% (11) | 6.3% (11) | 3.4%  (2) | 7.8%  (9) | 7.8% (9) | 0.271 | 0.03, 0.13 | 0.271 | 0.03, 0.13 |
| Hematological disease  % (n) iii | | 11.5% (20) | 13.2% (23) | 20.7%  (12) | 6.9%  (8) | 9.5%  (11) | 0.007 | 0.02, 0.12 | 0.040 | 0.02, 0.12 |
| Malignancy  % (n) | | 12.1% (24) | 12.1% (21) | 12.1%  (7) | 12.1%  (14) | 12.1%  (14) | 1 | 0.06, 0.18 | 1 | 0.06, 0.18 |
| Endocrinological condition (other than diabetes mellitus)  % (n) iv | | 14.9% (26) | 16.7% (29) | 31.0%  (18) | 6.9%  (8) | 9.5%  (11) | <0.001 | 0.02, 0.12 | <0.001 | 0.02, 0.12 |
| Diabetes Mellitus  % (n) | | 18.4% (32) | 19.0% (33) | 17.2%  (10) | 19.0%  (22) | 19.8%  (23) | 0.782 | 0.12, 0.26 | 0.682 | 0.12, 0.26 |
| Obesity  % (n) v | | 12.1% (21) | 14.4% (25) | 19.0%  (11) | 8.6%  (10) | 12.1%  (14) | 0.048 | 0.03, 0.14 | 0.221 | 0.03, 0.14 |
| Neurological disease  % (n) vi | | 23.0% (40) | 24.7% (43) | 37.9%  (22) | 15.5%  (18) | 18.1%  (21) | 0.001 | 0.09, 0.22 | 0.004 | 0.09, 0.22 |
| Psychiatric disorders  % (n) vii | | 18.4% (32) | 19.0% (33) | 25.9%  (15) | 14.7%  (17) | 15.5%  (18) | 0.072 | 0.08, 0.21 | 0.101 | 0.08, 0.21 |
| Chronic infectious disease  % (n) viii | | 8.0% (14) | 8.0% (14) | 10.3%  (6) | 6.9%  (8) | 6.9%  (8) | 0.431 | 0.02, 0.12 | 0.431 | 0.02, 0.12 |
| Smoking  % (n) | | 24.7% (43) | 24.7% (43) | 6.9%  (4) | 33.6%  (39) | 33.6%  (39) | <0.001 | 0.25, 0.42 | <0.001 | 0.25, 0.42 |
| Other disease  % (n) ix | | 33.9% (59) | 35.1% (61) | 37.9%  (22) | 31.9%  (37) | 33.6%  (39) | 0.428 | 0.23, 0.4 | 0.574 | 0.23, 0.40 |

i- Includes Hematology-Oncology, Otolaryngology (1 patient – without surgical intervention), Cardiac Intensive Care Unit, Cardiology, neurology.
ii- Includes Otolaryngology/ Head and Neck surgery, Orthopedics, Vascular surgery, Urology.
iii- Includes anemia, polycythemia, thalassemia, coagulation disorders, hematological malignancy.
iv- Includes hyper/hypo-thyroidisim, hyper/hypo-parathyroidism, osteoporosis, Addisson disease, Polycystic ovary syndrome, Cushing syndrome, panhypopituitirism.
v- Based on International Clinical Diseases-9 diagnosis code.
vi- Neuromuscular diseases, epilepsy, dementia, stroke, arterio-venous malformation, migranes.
vii- Autism, schizophrenia, schizo-affective disorder, depression, drug and alcohol abuse.
viii- Hepatitis B, hepatitis C, HIV, recurrent infections, Carbapenem Rresistent Klabsiella Pneumonia / Vancomycon Resistent Enterococcus carriers.
ix- Rheumatology disorders, inflammatory bowel disease, genetic disorders, underwent major surgeries, allergies, gastrointestinal conditions, immune deficiencies, pregnancy.

Statistically significant findings (p≤0.05) are highlighted. The 95% CI refers to the difference between proportions.

**Supplement 4b:** Pre-admission treatment with psychiatric drugs among among critically ill patients with intellectual disability versus matched patients without intellectual disability

|  | **Study group with original controls**  **(n=174) % (n)** | **Study group with modified controls**  **(n=174) % (n)** | **Study group**  **(n=58) % (n)** | **Original Controls**  **(n=116) % (n)** | **Modified controls (n=116) % (n)** | **p-Value** vs. original controls | **95% CI** | **p-Value** vs. modified controls | **95% CI** |
| --- | --- | --- | --- | --- | --- | --- | --- | --- | --- |
| Yes | 25.9% (45) | 27.6% (48) | 53.4%  (31) | 12.1%  (14) | 14.7%  (17) | **<0.001** | 0.06, 0.18 | **<0.001** | 0.06, 0.18 |
| Antidepressants | 5.7% (10) | 5.7% (10) | 12.1%  (7) | 2.6%  (3) | 2.6%  (3) | **0.017** | 0, 0.06 | **0.017** | 0, 0.06 |
| Typical antipsychotics | 4.6% (8) | 4.6% (8) | 13.8%  (11) | 0%  (0) | 0%  (0) | **<0.001** | 0, 0 | **<0.001** | 0, 0 |
| Atypical antipsychotics | 10.3% (18) | 10.3% (18) | 20.7%  (12) | 5.2%  (6) | 5.2%  (6) | **0.002** | 0.01, 0.09 | **0.002** | 0.01, 0.09 |
| Benzodiazepines | 10.3% (18) | 10.3% (18) | 20.7%  (12) | 5.2%  (6) | 5.2%  (6) | **0.002** | 0.01, 0.09 | **0.002** | 0.01, 0.09 |
| Mood stabilizers | 0.6% (1) | 0.6% (1) | 0%  (0) | 0.9%  (1) | 0.9%  (1) | 1 | 0, 0 | 1 | -0.01, 0.03 |
| Barbiturates | 1.1% (2) | 1.1% (2) | 3.4%  (2) | 0%  (0) | 0%  (0) | 0.110 | 0, 0 | 0.110 | 0, 0 |
| Anti-convulsants | 13.2% (23) | 15.5% (27) | 25.9%  (15) | 6.9%  (8) | 10.3%  (12) | **<0.001** | 0.02, 0.12 | **0.008** | 0.02, 0.12 |
| Anticholinergics | 3.4% (6) | 3.4% (6) | 8.6%  (5) | 0.9% (1) | 0.9% (1) | **0.008** | -0.01, 0.03 | **0.008** | -0.01, 0.03 |
| Stimulants / psychoactives | 0.6% (1) | 0.6% (1) | 1.7%  (1) | 0%  (0) | 0%  (0) | 0.333 | 0, 0 | 0.333 | 0, 0 |

Statistically significant findings (p≤0.05) are highlighted. The 95% CI refers to the difference between proportions.

**Supplement 5:** intubation characteristics and the weaning process among critically ill patients with intellectual disability versus matched patients without intellectual disability

|  | **Study group**  **(n=58)** | **Original controls**  **(n=116)** | **Modified controls (n=116)** | **p-Value**  vs. original controls | **95% CI** | **p-Value**  vs.  modified controls | **95% CI** |
| --- | --- | --- | --- | --- | --- | --- | --- |
| Proportion of patients intubated (within the full cohort)  % (n) | 86.2%  (50/58) | 42.2%  (49/116) | 37.9%  (44/116) | **<0.001** | 0.33, 0.51 | **<0.001** | 0.77, 0.95 |
| Time from intubation to first weaning attempt (days)  Mean$\pm SD$  [median; IQR; min-max] | 4.8±5.04  [1; 1-7.5; 0-23] | 3.6±3.4  [1; 1-6; 0-15] | 2.7±2.8  [1; 1-4; 0-11] | 0.177 | -0.57, 3.04 | **0.020** | 0.34, 3.8 |
| Time from first weaning attempt to extubation (days)  Mean$\pm SD$  [median; IQR; min-max] | 1.5±2.5  [1; 0-2; 0-14] | 0.5±1.4  [1; 0-1; 0-14] | 0.48±1.4  [1; 0-1; 0-14] | **0.010** | 0.24, 1.73 | **0.007** | 0.3, 1.78 |
| Intubation duration (days)  Mean$\pm SD$  [median; IQR; min-max] | 7.3±6.2  [5; 2-11; 1-24] | 5±3.5  [4; 2-7; 1-16] | 4.06±2.7  [4; 2-5; 1-12] | **0.038** | 0.13, 4.67 | **0.005** | 1.05, 5.45 |
| Number of extubation attempts Mean$\pm SD$  [median; IQR; min-max] | 1.1±0.6  [3; 1-1; 0-3] | 0.9±0.4  [3; 1-1 ;0-2] | 0.9±0.4  [3; 1-1 ;0-2] | 0.061 | -0.01, 0.42 | 0.093 | -0.03, 0.41 |
| Number of weaning attempts  Mean$\pm SD$  [median; IQR; min-max] | 1.19±0.890  [3; 1-2; 0-4] | 0.86±0.500  [3; 1-1; 0-2] | 0.88±0.498  [3; 1-1; 0-2] | **0.035** | 0.024, 0.64 | 0.055 | -0.007, 0.62 |
| Number of self extubations  Mean$\pm SD$  [median; IQR; min-max] | 0.05±0.221  [0; 0-1; 0-1] | 0.06±0.233  [0; 0-1 ;0-1] | 0.06±0.233  [0; 0-1; 0-1] | 0.7801 | -0.01, 0.12 | 0.7801 | -0.01, 0.12 |
| Proportion of ultimately  successful extubations  % (n) | 61.2%  (30) | 77.6%  (38) | 75.0%  (33) | 0.08 | 0.65, 0.89 | 0.156 | 0.65, 0.9 |
| Reintubation within 24 hours from extubation  % (n) | 5.2%  (3) | 3.4%  (4) | 3.4%  (4) | 0.592 | 0.12, 0.37 | 0.592 | 0, 0.16 |
| Tracheostomy (all patients)  % (n) | 20.7%  (12) | 2.6%  (3) | 2.6%  (3) | **<0.001** | -0.1, 0.13 | **<0.001** | -0.1, 0.13 |
| Tracheostomy (intubated patients)  % (n) | 24%  (12) | 6.1%  (3) | 6.1%  (3) | **0.005** | -0.1, 0.13 | **0.013** | -0.1, 0.13 |

The data on extubation of 1 patient was missing (transferred to another hospital while intubated)
Statistically significant differences (p<0.05) are highlighted. The 95% CI refers to the difference between proportions.

Supplement 6a: Medications given 48 hours before extubation among critically ill patients with intellectual disability versus matched patients without intellectual disability

|  | **Study group (n=58)** | **Original controls (n=116)** | **Modified controls (n=116)** | **p-Value**  vs. original controls | **95% CI** | **p-Value**  vs.  modified controls | **95% CI** |
| --- | --- | --- | --- | --- | --- | --- | --- |
| Total number of drugs given within 0-48 hours prior to extubation. Mean$\pm SD$  [median; IQR; min-max] | 2.8±1.1  [12; 2-4; 1-6] | 3.4±1.4  [14; 2-4.75; 1-6] | 3.2±1.5  [12; 2-4; 1-6] | 0.074 | -1.07, 0.05 | 0.192 | -0.06, 0.2 |
| Propofol  % (n) | 56%  (28) | 75.4%  (43) | 77.6%  (38) | **0.034** | 0.02, 0.56 | **0.023** | 0.02, 0.56 |
| Midazolam % (n) | 10%  (5) | 24.6%  (14) | 22.4%  (11) | **0.049** | -0.07, 0.35 | 0.092 | -0.06, 0.35 |
| Dexmedetomidine % (n) | 8%  (4) | 8.8%  (5) | 6.1%  (3) | 1 | 0, 0 | 1 | 0, 0 |
| Morphine % (n) | 8%  (4) | 19.3%  (11) | 20.4%  (10) | 0.093 | -0.07, 0.35 | 0.076 | -0.07-0.35 |
| Fentanyl % (n) | 12%  (6) | 26.3%  (15) | 20.4%  (10) | 0.063 | -0.07, 0.35 | 0.256 | -0.07, 0.35 |
| Remifentanil % (n) | 40%  (20) | 40.4%  (23) | 44.9%  (22) | 0.971 | -0.07, 0.35 | 0.622 | -0.07, 0.35 |
| Ketamine % (n) | 0%  (0) | 5.3%  (3) | 1%  (2) | 0.246 | 0, 0 | 0.495 | 0, 0 |
| Methadone % (n) | 14%  (7) | 19.3%  (11) | 22.4%  (11) | 0.465 | 0, 0 | 0.276 | 0, 0 |
| Seroquel % (n) | 8%  (4) | 19.3%  (1) | 18.4%  (9) | 0.093 | 0, 0 | 0.127 | 0, 0 |
| Clonex % (n) | 6%  (3) | 21.1%  (12) | 24.5%  (12) | **0.025** | 0, 0 | **0.010** | 0, 0 |
| Halidaol % (n) | 6%  (3) | 15.8%  (9) | 16.3%  (8) | 0.109 | 0, 0 | 0.102 | 0, 0 |
| Clozapine % (n) | 4%  (2) | 0% (0) | 0% (0) | 0.216 | -0.08, 0.22 | 0.495 | 0.08, 0.22 |

Statistically significant findings (p≤0.05) are highlighted. The 95% CI refers to the difference between proportions.

supplement 6b: Total dose of drugs given in the 48 hours before extubation among critically ill patients with intellectual disability versus matched patients without intellectual disability

|  | **Study group**  **(n=58)** | **Original controls (n=116)** | **Modified controls (n=116)** | **p-Value**  vs.  original controls | **95% CI** | **p-Value**  vs.  modified controls | **95% CI** |
| --- | --- | --- | --- | --- | --- | --- | --- |
| **IV continuous drugs mg/kg/hr** | | | | | | | |
| Propofol 0-24 hours prior to extubation, Mean$\pm SD$  [median; IQR; min-max] | 1.5±1  [1.5 ;0.6-1.9; 0.3-3.3] | 2.1±1.1  [1.9; 1.5-2.6; 0.4 -6.2] | 2.1±1  [1.9; 1.4-2.3; 0.4-6.2] | **0.033** | 1.72, 2.46 | **0.034** | 1.72, 2.4 |
| Propofol 24-48 hours prior to extubation, Mean$\pm SD$  [median; IQR; min-max] | 1.4$\pm$0.6  [1.5; 0.8-1.9; 0.4-2.4] | 2.1±1.9  [1.5;1.2-2.8; 0.4-4.9] | 2.1±1.1  [2.1; 1.2-2.5; 0.4-4.9] | **0.012** | 1.62, 2.62 | **0.009** | 1.7, 2.6 |
| Midazolam 0-24 hours prior to extubation, Mean$\pm SD$  [median; IQR; min-max] | None given | 0.1±0.1  [0.1; 0.03-0.2; 0.03-0.3] | 0.12±0.1  [0.09; 0.04-0.2; 0.03-0.33] | 0.857 | -0.003, 0.23 | 0.889 | 0.04-0.21 |
| Midazolam 24-48 hours prior to extubation, Mean$\pm SD$  [median; IQR; min-max] | None given | 0.08±0.07  [1.5 ;0.03  -0.1; 0.02-0.2] | 0.08±0.07  [0.05; 0.03  -0.1; 0.02-0.2] | 0.571 | 0.006, 0.15 | 0.571 | 0.005, 0.15 |
| Morphine 24-48 hours prior to extubation, Mean$\pm SD$  [median; IQR; min-max] | None given | 0.13±0.1  [0.13; 0.06-0.13; 0.07-0.2] | None given | 1 | -0.7,1 |  |  |
| Fentanyl 0-24 hours prior to extubation, Mean$\pm SD$  [median; IQR; min-max] | 0.02±0.04  [0.001;0.0007-0.05; 0-0.1] | 0.1±0.33  [0.001; 0.001-  0.003; 0-1.05] | 0.02±0.28  [0.002; 0.0008-  0.003; 0-1.05] | 0.594 | -0.13, 0.34 | 0.672 | -0.05, 0.26 |
| Fentanyl 24-48 hours prior to extubation, Mean$\pm SD$  [median; IQR; min-max] | 0.06±0.1  [0.002; 0.001-0.003; 0-0.2] | 0.003±0.001  [0.003; 0.002  -0.004; 0-0.1] | 0.003±0.001  [0.003; 0.001  -0.003; 0-0.1] | 1 | 0.001, 0.004 | 1 | 0.002, 0.004 |
| Remifentanil 0-24 hours prior to extubation, Mean$\pm SD$  [median; IQR; min-max] | 0.01 $\pm$0.02  [0.006; 0.003  -0.009; 0-0.08] | 0.01±0.01  [0.007; 0.005-0.009; 0-0.06] | 0.01±0.01  [0.008; 0.004-  0.01; 0-0.06] | 0.43 | 0.004, 0.01 | 0.331 | 0.005, 0.015 |
| Remifentanil 24-48 hours prior to extubation, Mean$\pm SD$  [median; IQR; min-max] | 0.006±0.003  [0.007;0.005  -0.001; 0-0.01] | 0.007±0.003  [0.007; 0.006-  0.01; 0-0.01] | 0.008±0.003  [0.007; 0.005-  0.01; 0-0.01] | 0.516 | 0.006,0.009 | 0.516 | 0.006,0.009 |
| Dexmedetomidine 24-48 hours prior to extubation, Mean$\pm SD$  [median; IQR; min-max] | None given | 0.35±0.14 [0.35; 0.5-0.6; 0.25-0.45] | 0.4±0.13 [0.4  ;0.2-0.4; 0.3-0.5] | 0.667 | -0.95,1.64 | 0.5 | -0.95,1.64 |
| Dexmedetomidine 0-24 hours prior to extubation, Mean$\pm SD$  [median; IQR; min-max] | 0.6±0.3  [0.6; 0.35-0.9; 0.3-1] | 0.6±0.08  [0.6; 0.2-0.35; 0.5-0.6] | 0.6±0.1  [0.6; 0.5-0.7; 0.5-0.7] | 1 | 0.36,0.76 | 1 | 0.49,0.69 |

| **Drugs given by bolus, mg** | | | | | | | |
| --- | --- | --- | --- | --- | --- | --- | --- |
| Methadone 0-24 hours prior to extubation, Mean$\pm SD$  [median; IQR; min-max] | 44±36.5  [30; 15-80;  10-100] | 48.3±43.4  [30; 20-90;  5-120] | 48.3±43.4  [30; 12.5-50;  5-120] | 0.898 | 14.93, 81.72 | 0.898 | 14.93, 81.72 |
| Methadone 24-48 hours prior to extubation, Mean$\pm SD$  [median; IQR; min-max] | 48±35.8  [60; 10-80;  10-85] | 70±36.5  [60; 40-120;  30-120] | 70±36.5  [60; 32.5-67.5;  30-120] | 0.530 | 36.22, 103.8 | 0.530 | 36.22, 103.77 |
| Clonex 0-24 hours prior to extubation, Mean$\pm SD$  [median; IQR; min-max] | 0.91±0.63  [1; 0.25-1;  0.25-1.5] | 1.9±1.5 [1; 0.75-2.5; 0.5-6] | 1.9±1.7  [1; 0.75-1.9; 0.5-6] | 0.209 | 0.66, 3.27 | 0.209 | 0.66, 3.27 |
| Clonex 24-48 hours prior to extubation, Mean$\pm SD$  [median; IQR; min-max] | None given | 2.9$\pm$1.7  [2.5; 1.5-4;  1.5-6] | 2.9±1.7  [2.5; 1.5-4;  1.5-6] | 0.286 | 1.11, 4.71 | 0.286 | 1.11,  4.71 |
| Quetiapine 0-24 hours prior to extubation, Mean$\pm SD$  [median; IQR; min-max] | 187.5±176.2 [112.5; 81.25- 368.75;  75-450] | 77.77±36.3  [75; 50-100;  25-150] | 72.7±36.1  [75 ; 37.5-93.8; 25-150] | 0.106 | 49.85, 5.69 | 0.078 | 48.44, 97.01 |
| Quetiapine 24-48 hours prior to extubation, Mean$\pm SD$  [median; IQR; min-max] | 133.8±86.9  [112.5; 63.75-225; 60-250] | 65±51.8  [50; 25-112.5;  25-150] | 58.9±47.2  [50; 15.6-75;  12.5-150] | 0.190 | 0.63, 129.36 | 0.109 | 15.3, 102.55 |
| Clozapine 0-24 hours prior to extubation, Mean$\pm SD$  [median; IQR; min-max] | 500±141.4  [500; 400-600; 400-600] | None given | None given |  |  |  |  |
| Clozapine 24-48 hours prior to extubation, Mean$\pm SD$  [median; IQR; min-max] | 500$\pm$141.4  [500; 400-600; 400-600] | None given | None given |  |  |  |  |
| Halidol 0-24 hours prior to extubation, Mean$\pm SD$  [median; IQR; min-max] | 3.5±2.1  [3.5 ,2-3.5;  2-5] | 20±21.7  [10; 5-37.5;  5-60] | 20±21.7  [10; 5-37.5; 5-60] | 0.143 | -2.75, 42.75 | 0.143 | -2.75, 42.75 |

Statistically significant findings (p≤0.05) are highlighted. The 95% CI refers to the difference between proportions.

Supplement 7: Admission characteristics and mortality rates among critically ill patients with intellectual disability versus matched patients without intellectual disability

|  | **Study group**  **(n=58)** | **Original controls (n=116)** | **Modified controls (n=116)** | **p-Value**  vs.  original controls | **95% CI** | **p-Value**  vs.  modified controls | **95% CI** |
| --- | --- | --- | --- | --- | --- | --- | --- |
| Overall hospital admission duration (days)  Mean$\pm SD$  [median; IQR; min-max] | 30.7±40.3  [20; 8.8-36  ; 0-230] | 18.5±18.3  [24 ;7.3-  22; 2-90] | 16.4±16.9  [24 ;6.3-  19.8; 2-90] | **0.019** | 2.25, 24.1 | **0.032** | 1.07, 23.2 |
| ICU admission duration (days)  Mean$\pm SD$  [median; IQR; min-max] | 11±11.4  [5.5; 3-16  ; 0-60] | 5.6±4.2  [6; 2-7; 1-22] | 4.9±3.7  [6; 2-7; 1-18] | **0.001** | 2.46, 8.59 | **0.001** | 2.24, 8.41 |
| Second admissions to the hospital with ICU admission Mean$\pm SD$  [median; IQR; min-max] | 0.4±0.7  [1; 0-1; 0-3] | 0.1±0.46  [1; 0-0; 0-3] | 0.2±0.5  [1; 0-0; 0-3] | **0.024** | 0.006, 0.42 | **0.024** | 0.01, 0.43 |
| Readmissions to the ICU within the same hospital admission % (n) | 6.9%  (4) | 9.5%  (11) | 8.6%  (10) | **0.044** | 0.54, 1.03 | **0.036** | 0.54, 1.03 |
| Mortality in the ICU % (n) | 5.2%  (3) | 4.3%  (5) | 4.3%  (5) | 0.798 | -0.08­, 0.23 | 0.798 | -0.08­, 0.23 |
| Overall mortality (in the ICU and outside of the ICU)  % (n) | 19%  (11) | 9.5%  (11) | 9.5%  (11) | 0.076 | -0.07, 0.35 | 0.076 | -0.07-0.35 |
| Complication during ICU hospitalization  % (n) | 34.5%  (20) | 10.3%  (12) | 8.6%  (10) | **<0.001** | 0.07, 0.64 | **<0.001** | 0.07, 0.64 |
| cardiac complication during ICU hospitalization  % (n) | 3.4%  (2) | 0.9%  (1) | 0.9%  (1) | 0.217 | -0.08, 0.23 | 0.217 | -0.08, 0.23 |
| Infection complication during ICU hospitalization  % (n) | 29.3%  (17) | 4.3%  (5) | 2.6%  (3) | **<0.001** | -0.07, 0.35 | **<0.001** | -0.07, 0.35 |
| pulmonary complication during ICU hospitalization  % (n) | 8.6%  (5) | 1.7%  (2) | 1.7%  (2) | **0.029** | -0.07, 0.2 | **0.029** | -0.07, 0.35 |
| renal complication during ICU hospitalization  % (n) | 5.2%  (3) | 1.7%  (2) | 1.7%  (2) | 0.335 | 0, 0 | 0.335 | 0, 0 |
| neurological complication during ICU hospitalization  % (n) | 5.2%  (3) | 2.6%  (3) | 2.6%  (3) | 0.402 | -0.08­, 0.23 | 0.402 | -0.08­, 0.23 |
| sepsis complication during ICU hospitalization  % (n) | 6.9%  (4) | 0%  (0) | 0%  (0) | **0.012** | 0, 0 | **0.012** | 0, 0 |
| Pressure ulcer complication during ICU hospitalization  % (n) | 0%  (0) | 0%  (0) | 0%  (0) | Irrelevant | 0, 0 | Irrelevant | 0, 0 |
| Pulmonary embolism complication during ICU hospitalization  % (n) | 0%  (0) | 0%  (0) | 0%  (0) | Irrelevant | 0, 0 | Irrelevant | 0, 0 |
| DVT complication during ICU hospitalization  % (n) | 1.7%  (1) | 0%  (0) | 0%  (0) | 0.333 | 0, 0 | 0.333 | 0, 0 |

Rates were calculated from the entire group (n=174) with no missing data. Statistically significant differences (p<0.005) are highlighted. The 95% CI refers to the difference between proportions.

**Supplement 8:** Main diagnosis at the time of intensive care admission according to the International classification of Diseases-9, among critically ill patients with intellectual disability versus matched patients without intellectual disability.

|  | **Study group**  **(n=58)** % (n) | **Original controls**  **(n=116)** % (n) | **Modified controls**  **(n=116)** % (n) |
| --- | --- | --- | --- |
| Infectious and parasitic diseases | 51.7% (30) | 32.8% (38) | 35.3% (41) |
| Diseases of the blood and blood-forming organs | 1.7 (1) | 1.7 (2) | 2.6 (3) |
| Diseases of the circulatory system | 3.4 (2) | 2.6 (3) | 3.4 (4) |
| Diseases of the digestive system | 12.1 (7) | 6.9 (8) | 12.1 (14) |
| Diseases of the genitourinary system | 0 (0) | 3.4 (4) | 3.4 (4) |
| Diseases of the nervous system and sense organs | 20.7 (12) | 16.4 (19) | 24.1 (28) |
| Diseases of the respiratory system | 0 (0) | 1.7 (2) | 1.7 (2) |
| Endocrine, nutritional, and metabolic diseases, and immunity disorders | 3.4 (2) | 2.6 (3) | 5.2 (6) |
| Injury and poisoning | 6.9 (4) | 27.6 (32) | 6.9 (8) |
| Complications of pregnancy, childbirth, and the puerperium | 0 (0) | 4.3 (5) | 5.2 (6) |
